# Lack of Consensus on the Definition of Aseptic Loosening in Total Ankle Replacement: A Narrative Systematic Review

**DOI:** 10.3390/jcm13030786

**Published:** 2024-01-30

**Authors:** Peter Kvarda, Andreea Mills, David Shepherd, Tim Schneider

**Affiliations:** 1 Melbourne Orthopaedic Group, 33 The Avenue, Windsor, Melbourne, VIC 3181, Australia; 2 Royal Australasian College of Surgeons, Victoria State Office, 250-290 Spring Street, Melbourne, VIC 3002, Australia

**Keywords:** aseptic loosening, total ankle replacement, failure, definition, consensus

## Abstract

**Background:** Aseptic loosening is one of the most common modes of failure of total ankle replacement (TAR). However, a precise definition of aseptic loosening is still lacking. This systematic review aimed to identify the variations of applied definitions and offer insights into the lack of consensus. **Methods:** Human studies reporting aseptic loosening of TAR published in peer-reviewed journals within the last decade were considered. The search strategy involved specific terms in Embase, MEDLINE ALL, and the Cochrane Library. Variations in aseptic loosening definitions were analysed. **Results:** Of 767 studies, 88 were included in this study. Only nine studies precisely defined aseptic loosening with significant variations. Twenty-two studies referenced the term and fifty-seven reported it as a complication but neither defined nor referenced it. **Conclusions:** Significant uncertainty exists regarding the universal definition of aseptic loosening of TAR, and many variations occur in terms of the assessment approach and criteria.

## 1. Introduction

Total ankle replacement (TAR) is a surgical procedure that has been increasingly utilized as a treatment option for end-stage ankle arthritis, offering pain relief and improved joint function [1]. However, aseptic loosening remains a significant concern as one of the most common complications associated with TAR [2,3,4]. It is characterized by the dissociation of the implant–bone interface in the absence of infection, leading to instability, pain, and functional impairment [5,6]; moreover, it can require revision surgery and adversely affect the long-term outcomes of TAR [7,8,9]. The pathomechanism of aseptic loosening of total ankle components has been studied in detail, including polyethylene debris-induced osteolysis, micromotion at the bone–implant interface, high joint fluid pressure, implant size, and possible genetic factors [6].

To effectively diagnose and manage aseptic loosening of TAR, it is crucial to establish a clear and consistent definition. However, the current literature reveals a lack of consensus among researchers and clinicians regarding the specific definition of aseptic loosening of TAR. This lack of consensus raises concerns regarding diagnostic accuracy and outcome reporting.

In delving into the literature gap concerning the absence of consensus on the definition of aseptic loosening in TAR, it becomes apparent that several factors may contribute to this ambiguity. The author’s opinion on this matter stems from a combination of inherent complexities in ankle biomechanics, the evolving nature of surgical techniques and implant designs, and the diverse range of criteria used in different studies. Firstly, the complexity of ankle joint anatomy and biomechanics poses a unique challenge compared to other weight-bearing joints. Unlike hip or knee replacements, where consensus has been relatively easier to achieve, the ankle morphology and anatomy, which often change posttrauma, demand a more thorough understanding. Secondly, the field of ankle replacement surgery is continually evolving. New implant designs, surgical approaches, and fixation methods are being introduced, contributing to a lack of standardization in assessing aseptic loosening. The ongoing refinement of surgical techniques and the introduction of innovative materials/designs may influence the longevity and stability of ankle implants, further complicating efforts to establish a consensus on defining aseptic loosening. Additionally, the relatively limited long-term follow-up data for total ankle replacement may contribute to the lack of consensus as time is required for complications such as aseptic loosening to manifest. Moreover, the heterogeneity in assessment criteria employed across different studies exacerbates the challenge of achieving consensus. Variability in diagnostic modalities, such as radiographic assessments, clinical evaluations, and functional outcomes, makes it difficult to compare findings directly. 

In summary, the lack of consensus on the definition of aseptic loosening in total ankle replacement appears to be a multifaceted issue. Addressing this gap necessitates collaborative efforts within the research community to establish standardized definitions that can accommodate the evolving landscape of ankle replacement surgery while providing meaningful and comparable outcomes across studies and to bridge this crucial gap in the literature.

This narrative systematic review aimed to comprehensively analyse the available literature to identify variations and discrepancies in the definitions used and to examine the potential implications of the lack of consensus on the definition of aseptic loosening in TAR. 

## 2. Materials and Methods

### 2.1. Eligibility Criteria

Inclusion criteria were as follows: studies reporting aseptic loosening in the context of total ankle replacement, studies involving human participants, and studies published in peer-reviewed journals. There were no restrictions on study design, applied surgical technique, implant design, or baseline characteristics of the cohort. Studies published in the last decade were considered.

Focusing on the last decade allows us to consider the most recent and pertinent literature on the subject. Recent scientific papers are likely to incorporate the latest and most precise methodologies and information. Since the early years of the second decade of this century, there has been an exponential growth in research and publications in the field. Throughout this period, the majority of publications have centered on well-established third-generation prosthetic designs, with some also addressing more contemporary fourth-generation designs. Notably, this approach potentially excludes earlier first- and second-generation designs known for significant technical and design flaws, reflecting the developing scientific profile of the field during that time. Contemporary orthopedic surgeons show a heightened interest in current scientific findings. Moreover, older studies are susceptible to publication bias, where positive results are selectively published. By restricting the search to the last decade, we aim to mitigate this bias. This limitation not only enhances the efficiency of our systematic review but also streamlines the time-consuming process of conducting the review, while capturing a substantial body of literature.

Animal studies, conference abstracts, technical notes, system design descriptions, letters to the editor, and non-English-language studies were excluded. Studies published prior to 2013 were excluded.

**Figure 1:**
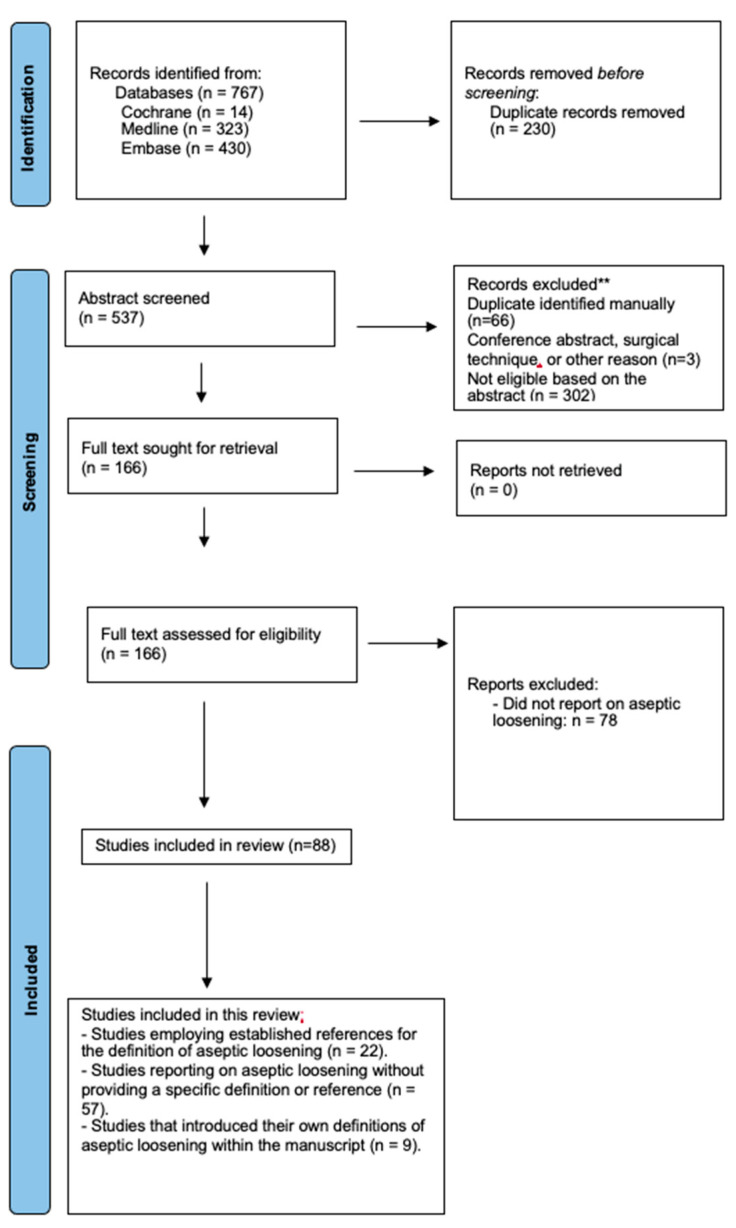
PRISMA flow diagram.

Methodological considerations: studies using references to other studies for the definition of aseptic loosening of the TAR were included in our analysis; however, these studies were noted separately. This study adhered to the Preferred Reporting Items for Systematic Review and Meta-Analysis Protocols (PRISMA-P) guideline guidelines [10] (Figure 1). A preliminary search was conducted on the 14 June 2023. The study was officially registered on the International Platform of Registered Systematic Review and Meta-Analysis Protocols (INPLASY) on the 14 June 2023 (Registration Number: INPLASY202360038) [11,12]. The research question was formulated using the Population, Intervention, Comparison, Outcome Study design (PICOS) tool [13] (Table 1). 

Furthermore, this study was conducted to fulfill the requirements outlined in the Scale for the Assessment of Narrative Review Articles [14].

### 2.2. Information Sources and Search Strategy

A combination of text words, synonyms, and variations, along with database-specific subject headings, was used to conduct the search. The electronic databases Embase via Embase.com, Medline ALL via Ovid, and the Cochrane Library were searched. The retrieved references were exported to the Mendeley citation manager, and duplicates were removed. In addition to electronic database searches, the bibliographic references and citations of all the included articles were screened to identify additional relevant studies that may have been missed. The initial search was conducted on the 1 July 2023. 

The following search strategy was developed and applied: 

Database: Ovid MEDLINE^®^ ALL.

((exp Arthroplasty, Replacement, Ankle/OR (ankle* adj3 (arthroplast* or replace*)).ti,ab,kw.) AND (exp Prosthesis Failure/OR exp Equipment Failure Analysis/OR (aseptic adj3 loose*).ti,ab,kw. OR (prosthe* adj3 (fail* or loose*)).ti,ab,kw. OR (exp Radiography/AND exp Treatment Outcome/) OR (radiograph* adj3 (result* or outcome* or assess*)).ti,ab,kw.)) NOT (comment OR letter OR “systematic review”).pt. 

Database: Embase.

((exp ankle arthroplasty/OR exp ankle replacement/OR exp ankle prosthesis/OR (ankle* adj3 (arthroplast* OR replace*)).ti,ab,kw.) AND (exp prosthesis complication/OR exp device failure analysis/OR exp prosthesis loosening/OR (aseptic adj3 loose*).ti,ab,kw. OR (prosthe* adj3 (fail* or loose*)).ti,ab,kw. OR ((exp radiography/OR exp ankle radiography/) AND (exp treatment outcome/OR exp clinical outcome/)) OR (radiograph* adj3 (result* or outcome* or assess*)).ti,ab,kw.)) NOT (conference abstract/or “systematic review”/or letter/or (conference-abstract or letter).pt).

Cochrane Library.

https://www.cochranelibrary.com/advanced-search/search-manager (accessed on 1 July 2023).

([mh “Arthroplasty, Replacement, Ankle”] OR ((ankle*) NEAR/3 (arthroplast* or replace*)):ti,ab,kw) AND ([mh “Prosthesis Failure”] OR [mh “Equipment Failure Analysis”] OR ((aseptic) NEAR/3 (loose*)):ti,ab,kw OR ((prosthe*) NEAR/3 (fail* or loose*)):ti,ab,kw OR ([mh “Radiography”] AND [mh “Treatment Outcome”]) OR ((radiograph*) NEAR/3 (result* or outcome* or assess*)):ti,ab,kw).

### 2.3. Data Extraction and Data Items

Two reviewers (P.K. and T.S.) initially screened in a tabular form the retrieved references, using the abovementioned search strategy, based on titles and abstracts to identify studies that potentially met the inclusion criteria. Any relevant references were retrieved from the full text and independently assessed by the same two reviewers (P.K. and T.S.). In case of any disagreement regarding study eligibility, a consensus was reached through discussion by the same authors (P.K. and T.S.). If needed, a third author (D.S.) made the final decision. Data from full-text articles were extracted, entered into a standardized tabular form, and summarized in a narrative synthesis. The primary variable used in the reviewed studies was the definition of aseptic loosening. Secondary variables included study characteristics such as year of publication, country of study, study design, publishing journal, sample size, applied implant design, follow-up time, and imaging modality. Data extraction was performed by two authors (P.K. and T.S.). In any case of disagreement on relevant data to be extracted, a consensus was similarly reached as previously mentioned by the same authors (P.K. and T.S.). If necessary, a third author (D.S.) made the final decision.

## 3. Results

### 3.1. Study Selection

The initial search yielded 767 studies. After removing duplicates and excluding ineligible studies, 166 full-text articles were screened. Seventy-eight studies were excluded based on further assessment and lack of reporting on aseptic loosening. A total of 88 studies were included in this review (Figure 1).

### 3.2. Study Characteristics

The baseline characteristics of the final included studies are presented in Table 2.

### 3.3. Applied Definitions in Included Studies

The various definitions applied in the included studies are presented in Table 3.

### 3.4. References Utilized for the Definition of Aseptic Loosening

The number of papers included where aseptic loosening was reported as a complication but was neither defined nor referenced was 57 (65%). Table 4. presents the number of publication using references for defining aseptic loosening.

### 3.5. Quality Assessment

The quality of the individual studies defining aseptic loosening was independently assessed based on the Methodological Index for Non-randomized Studies (Minors) by two reviewers (PK and TS) (Table 5) [54].

## 4. Discussion

The lack of a consensus on the definition of aseptic loosening in TAR is still a significant challenge. In this systematic review, we aimed to comprehensively analyse the variations and discrepancies in the definitions of aseptic loosening in TAR. 

Although reliable and useful techniques have been developed for assessing other joint replacements, TAR systems lack universally accepted methods. 

Harris et al. attempted to categorize the different statuses of hip replacement loosening. According to the authors, a general rule can be applied in which component loosening is more likely to occur when lucent radiological lines exhibit a higher degree of progression. According to Mushtaq et al., stable 1–2 mm radiolucent lines can be interpreted as stable fibrous tissue ingrowth [55]. Attempts have been made with different approaches to assess component migration and loosening of TAR. Brigido et al. described their technique of measuring the complete length of the investigated stemmed design implants in conventional radiographs [56]. However, this technique lacks integration of bony references, which could provide reliable measurements of changes in component position [57]. Fong et al. reported their technique based on radiostereometric (RSA) analysis in vivo after marker insertion as reliable for assessing component micromotion [58]. A similar model-based RSA technique has been used by Dunbar et al. in assessing the micromotion of TAR, using a mobile-bearing design, based on longitudinal migration and inducible displacement measures. They concluded that the technique is capable of detecting pathological implant fixation [59]. Furthermore, RSA analysis was already adopted in hip and knee surgery in the early 1990s. Kärrholm et al. investigated 84 cemented hip replacements in the mid-term. They emphasized the determination of early micromotion of implant components, as it poses a risk of later need for revision. However, they also stated that the RSA technique due to its accuracy may not provide comparable measures for different implant designs. A larger group of 143 patients after knee replacement were evaluated using the same measurement technique by Ryd et al. These authors confirmed the applicability of this measurement technique and found that RSA evaluation can identify implants at risk with a predictive power of about 85% [60,61]. However, it has not been widely adopted in routine clinical practice.

While migration and subsidence can be stable, and the component might find firm osteointegration, aseptic loosening includes dynamic, unstable motion of the component. This dynamic component may not be visible on plain radiography or CT, where the affected limb is in a stationary position, making the task even more challenging. The question remains whether other imaging modalities, such as MRI or SPECT-CT, play a role in evaluating component loosening in TAR. A recent study by Endo et al. reported higher sensitivity than conventional radiographs in evaluating the loosening of total knee arthroplasty [62]. SPECT-CT has been shown to be a high-performance modality for total knee and hip arthroplasty in diagnosing loosening [63]. Our research identified only one study utilizing this advanced method, which can provide detailed information about the component fixation and osteointegrative process. Also, weight-bearing CT could presumably detect more subtle differences in component positions over time than two-dimensional radiographs. It is well known that quantification of the migration of TAR components poses challenges, primarily due to the absence of potential reference points and the possibility of morphological changes over time.

One of the main factors contributing to the lack of consensus is variability in the radiological criteria. Various studies have used different thresholds, such as the width of lucency, presence of subsidence, or migration of components. Not only do the measurements vary, but the follow-up duration between radiographs also does so. Specifically, variability in the follow-up duration also affects the ability to detect aseptic loosening, which may develop gradually over time. Different bone qualities and their different physiological reactions to the components and related varying speeds of osteointegration must be considered when determining the minimum follow-up duration.

Additionally, differences in assessment methods and follow-up durations have contributed to the lack of consensus. Some studies have relied solely on radiographic assessments, whereas others have incorporated clinical and functional outcomes. Interestingly, only one study included clinical signs as part of its definitions. In our opinion, clinical symptoms in accordance with radiological findings should always be included in the diagnosis. Further research should shed light on the typical clinical signs and features of aseptic loosening of the TAR, helping clinicians assess and differentiate it from symptoms of other origins. 

TAR involves various implant designs and surgical techniques. The diversity of implant designs and techniques introduces additional complexity to the evaluation of component migration and aseptic loosening. Different implant designs may have unique features and radiographic characteristics that require specific evaluation.

The strengths of this study include its comprehensive literature search, systematic approach, and objective analyses. However, this study has some limitations. First, it was limited to articles published in English, potentially introducing language bias. Studies published in other languages might have been overlooked. Second, systematic reviews may be susceptible to publication bias, as published studies tend to report significant findings or positive outcomes, whereas the non-publication of studies with null or negative results may impact the overall conclusions. Third, the definitions of aseptic loosening used in the included studies may vary in terms of terminology, criteria, and assessment methods, thereby introducing heterogeneity that may limit direct comparisons. Fourth, the review assessed the quality and risk of bias of the included studies; however, inherent limitations in the study design and methodology across the literature may influence the overall reliability and generalizability of the findings. Fifth, the definitions of aseptic loosening used in the included studies may vary in terms of terminology, criteria, and assessment methods, thereby introducing heterogeneity that may limit direct comparisons. Finally, because this study followed a narrative synthesis approach, it did not conduct a quantitative meta-analysis and may not provide pooled effect sizes or statistical estimates, limiting the ability to summarize findings across studies quantitatively.

## 5. Conclusions

Although TAR is a well-investigated field of foot and ankle surgery, there is still significant uncertainty regarding the universal definition of aseptic loosening of TAR, and many variations exist in terms of the assessment approach and criteria. Among the available works in the literature, we believe that the applied definition by Hintermann et al. is adequate and should be used as a starting point for further discussion [33]. However, we propose to establish thresholds for lucency width, presence of subsidence, migration of components, and standardized follow-up duration. The definition of aseptic loosening should be based on these. Publishing journals and regulatory bodies including national joint registries should also incorporate into their requirements the application of a standardized definition. 

Further research should strive to establish and incorporate comprehensive and standardized measures. This includes accounting for the bony integration process, determining the optimal follow-up duration, and exploring the utility of advanced imaging modalities in routine clinical practice. Our findings may encourage leading foot and ankle surgeons and researchers to establish a consensus.

## 6. Future Directions

Over the last decades, the number of articles published about outcomes of TAR has been continuously rising. Despite this upward trend, there is a lack of consensus and uniformity in reporting fundamental elements of these results. To address this issue in the future, efforts should be directed toward establishing an international consensus for the evaluation of TAR outcomes, regardless of implant design. Additionally, there is a need for journals specializing in orthopedics and related topics to mandate consistent and standardized assessment and communication of TAR results. This will contribute to greater coherence and reliability in the reporting of findings in the field.

## Figures and Tables

**Table 1:** The Population, Intervention, Comparison, Outcome, Study design (PICOS) tool.

Item	Specification
Population, or participants and conditions of interest	Patients with ankle OA
Interventions or exposures	Total ankle replacement
Comparisons or control groups	Not applicable
Outcomes of interest	Applied definition of aseptic loosening of TAR
Study designs	No restriction on study design. Only studies published in peer-reviewed journals.

**Table 2:** Baseline characteristics of studies defining aseptic loosening. USA: United States of America; n/a: not applicable; CT: computed tomography; SPECT-CT: single-photon emission computed tomography; STAR: Scandinavian Total Ankle Replacement.

Study	Year of Publication	Country of Study	Study Design	Publishing Journal	Sample Size	Applied Implant Design	Follow-Up Time	Imaging Modality	Outcome Measures Related to Aseptic Loosening
Kerkhoff et al. [15]	2016	The Netherlands	Retrospective case series	Foot and Ankle International	124	STAR	Minimum of 7.5 years	X-ray	n/a
Preis et al. [16]	2016	Germany	Retrospective case series	Clinical Orthopaedics and Related Research	18	HINTEGRA	mean 54 months	X-ray	n/a
Omar et al. [17]	2019	USA	Radiological descriptive	Seminars in Musculoskeletal Radiology	n/a	n/a	n/a	X-ray, CT	n/a
Marks et al. [18]	2019	USA	Prospective, multicenter, observational cohort	The Journal of Foot and Ankle Surgery	46	Salto Talaris	Mean 4.9 years ((range 0.9 to 8.6)	X-ray	n/a
Zafar et al. [19]	2020	Denmark	Retrospective case series	Acta Orthopaedica	322	HINTEGRA	Not described	X-ray	n/a
Gurbani et al. [20]	2020	USA	Retrospective case series	Foot and Ankle International	37	Not described	Mean 16.9 months (range 5–43)	SPECT-CT	n/a
Albagli et al. [21]	2021	Canada	Retrospective case series	Foot and Ankle Surgery	41	Infinity	mean 24 months	X-ray	n/a
Richter et al. [22]	2021	Switzerland	Retrospective case series	Clinical Orthopaedics and Related Research	935	HINTEGRA	Mean (range) 9 ± 4 years (2 to 17)	n/a (clinical definition)	n/a
Baumfeld et al. [23]	2022	Brazil	Retrospective case series	Foot and Ankle Surgery	29	Corin-Zennith	mean 5 years	X-ray	n/a

**Table 3:** Applied definitions of aseptic loosening in the included studies.

Study	Definition of Aseptic Loosening of TAR
Omar et al. [17]	Radiographically, loosening is determined when there is lucency around the prosthetic component at the prosthesis–bone interface that measures > 2 mm in width.
Preis et al. [16]	The criteria for radiographic loosening was defined as subsidence or migration of prosthesis components and/or a cystic lesion with a diameter at least 2 mm.
Baumfeld et al. [23]	Loosening of the tibial component was diagnosed when there were changes of more than two degrees in the alpha and beta angles, or when radiolucent lines of more than 2 mm in thickness appeared. Loosening of the talar component was diagnosed when deepening of the talus body greater than 5 mm occurred (distances “a” and “b”), or when the theta angle underwent changes greater than five degrees.
Albagli et al. [21]	Differences in alpha or beta angle of more than 2 degrees between the two timepoints and a radiolucent line of more than 2 mm around the component were indicative of undesired component motion. The talar component was assessed using the gamma angle as well as “a” and “b” distances measured on lateral radiographs. Differences in gamma angles of more than 5 degrees or a change in “a” or “b” values of more than 5 mm were indicative of undesired component motion and subsidence.
Richter et al. [22]	Aseptic loosening was defined as intra-operatively verified component loosening with or without periarticular cysts or avascular necrosis.
Zafar et al. [19]	Aseptic loosening was defined as the failure of the bond between an implant and bone, in the absence of infection, defined on radiographic findings of radiolucent lines around the implant and/or preoperatively as lack of bony ingrowth.
Gurbani et al. [20]	SPECT-CT images were interpreted using the following criteria. Strong activity that was evenly distributed beneath an implant was considered to be consistent with aseptic loosening.
Marks et al. [18]	Device loosening was defined as the presence of new or progressive radiolucency along the device–bone interface indicating a loss of fixation.
Kerkhoff et al. [15]	Radiographic loosening was defined as radiolucency of more than 2 mm which progressed over time.

**Table 4:** Included studies using references for the definition of aseptic loosening.

Nr. of Studies	Used Reference
Overall *n* = 22 (25%)	
1 [24]	Kim et al. [25]
8 [1,26,27,28,29,30,31,32]	Hintermann et al. [33]
1 [34]	Pugely et al. [35]
2 [36,37]	Trajkovski et al. [38]
1 [39]	Behrens et al. [40]
1 [41]	Kim et al. [42]
4 [43,44,45,46]	Lee et al. [47]
3 [48,49,50]	Wood et al. [51]
1 [52]	Schimmel et al. [53]

**Table 5:** Quality assessment of studies defining aseptic loosening. n/a: not applicable.

Study	Clearly Stated Aim	Inclusion of Consecutive Patients	Prospective Collection of Data	Endpoints Appropriate	Unbiased Assessment of Endpoints	Follow-Up Period Appropriate	Loss to Follow-Up Less Than 5%	Prospective Calculation Of Study Size	Overall Score
Omar et al. [17]	n/a	n/a	n/a	n/a	n/a	n/a	n/a	n/a	n/a
Preis et al. [16]	2	2	1	2	1	2	2	0	12
Baumfeld et al. [23]	1	2	1	2	0	2	0	0	8
Albagli et al. [21]	2	2	1	2	1	2	0	0	10
Richter et al. [22]	2	2	1	2	2	2	1	0	12
Zafar et al. [19]	1	2	1	2	1	2	0	0	9
Gurbani et al. [20]	2	2	1	2	1	2	0	0	10
Marks et al. [18]	2	2	2	2	1	2	2	0	13
Kerkhoff et al. [15]	2	2	2	2	1	2	0	0	11

## Data Availability

No new data were created in this study.
